# A qualitative exploration of student perceptions of the impact of progress tests on learning and emotional wellbeing

**DOI:** 10.1186/s12909-017-0984-2

**Published:** 2017-08-29

**Authors:** Jill Yielder, Andy Wearn, Yan Chen, Marcus A. Henning, Jennifer Weller, Steven Lillis, Vernon Mogol, Warwick Bagg

**Affiliations:** Faculty of Medical and Health Sciences, University of Auckland, Auckland, New Zealand

**Keywords:** Progess tests, Approaches to learning, Strategies for learning, Emotional wellbeing

## Abstract

**Background:**

Progress testing was introduced to the MBChB programme at the University of Auckland in 2013. As there has been a focus in published literature on aspects relating to the format or function of progress tests, the purpose of this study was to explore a qualitative student perspective on the introduction of progress testing and its impact on approaches to learning and perceived stress.

**Methods:**

This article presents the qualitative aspects of a longitudinal evaluation study. The qualitative data were derived from eight focus groups of Year 2–5 medical students in the University of Auckland medical programme.

**Results:**

Two themes, ‘Impact on Learning’ and ‘Emotional Wellbeing’ and their subthemes offered insight into student perceptions and behaviour. Students described a variety of learning responses to progress testing that clustered around the employment of a range of learning strategies based on their experience of sitting progress tests and their individualised feedback. A range of emotional responses were also expressed, with some finding progress tests stressful, while others enjoyed not needing to intensively cram before the tests.

**Conclusions:**

Progress tests appear to influence the approach of students to their learning. They employ a mix of learning strategies, shaped by their performance, individualised feedback and the learning environment. While students expressed some stress and anxiety with respect to sitting progress tests, this form of testing was viewed by these students as no worse, and sometimes better than traditional assessments.

## Background

Progress testing is used formatively and summatively in many medical schools. The rationale for progress testing is that it benefits students through fostering deep learning strategies, encouraging significant growth in applied medical knowledge through frequent testing and the use of feedback to enhance student progress [1]. It is also claimed that progress testing changes student study habits and reduces examination stress [2]. Progress tests require students to understand their learning in a clinical context, to integrate medical science with clinical practice and extend their knowledge of medical science into later years of the programme [3]. Progress testing has been described as an assessment ‘for learning’, rather than simply ‘of learning’, due to the way progress tests provide feedback for individual students. Student scores also inform curriculum performance and indications for change [4, 5]. With these goals in mind the University of Auckland medical programme introduced progress testing in 2013.

To date much of the focus on progress testing in the literature has been on the metrics of the format [6, 7]. Data shows that students do achieve higher scores over time using this format and lessons have been learnt about item construction and performance. The evidence on whether the approach changes learner behaviour is less clear. A fundamental tenet of progress testing is that it encourages students to learn consistently, deeply and to consolidate learning [4, 8].

What is currently lacking from the literature is a student perspective on the value and impact of progress testing. What is not known is whether students’ habits change and it is unclear whether progress testing drives the type of learning that we hope for. In an attempt to address these questions in our setting, a longitudinal research project was introduced in tandem with the introduction of progress testing. The study evaluated the first three years of implementation to ensure that the impact of this form of assessment on the students was understood. The overall study sought to explore the following questions:

1. What is the impact of progress testing on student approaches to learning?
2. What is the impact of progress testing on the integration of sciences and clinical practice across the programme?
3. What is the impact of progress testing on perceived stress associated with more frequent tests?
4. What are the unanticipated consequences of the introduction of progress testing?
5. How are the effects of progress testing on student approaches to learning and stress mitigated over time?

This article presents the findings of the qualitative part of the study, which focused on questions one, two and three. While this article discusses the qualitative results established from focus groups, where relevant, reference is made to the key results from the concurrent quantitative survey published separately [9]. The rationale for collecting qualitative data was to add richness and triangulation to the quantitative data.

## Methods

### Setting

As part of the re-design of our curriculum, progress testing was added as the primary assessment for applied medical knowledge in 2013. The medical programme consists of six years of study, with the first year being a common health sciences year. Students are selected from Year 1 into the MBChB, which begins in Year 2. The Year 2 and 3 curriculum (Phase 1) consists of integrated organ-system modules with some early clinical learning. In Years 4 to 6 (Phases 2 and 3), students rotate through clinical attachments in community and hospital practice. Progress testing begins in Year 2 and there are three tests per year. The test uses single best answer from five choices with formula scoring and a sixth ‘don’t know’ option.

### Design

The overarching research method for the study was programme evaluation. This approach normally assesses the rationale, effectiveness, efficiency, effects and impacts of an intervention or project/programme [10, 11].

The research reported in this article consisted of focus groups drawn from students in Years 2–5 of the programme during 2014, to explore the impact of the introduction of progress testing in more depth. For details of the quantitative survey also included as part of the overall study, please refer to Chan et al. [9].

The aim of the research was to determine students’ approaches to learning (deep/surface), as described by Biggs et al. [12], influences on approaches to learning, their associated stress levels, and how this changed over time. The qualitative data from the focus groups, as presented in this article, aimed to provide triangulation and depth to the overall study and enhance its internal validity [13, 14]. It was anticipated that the future use of progress testing could be optimised through an exploration of how the students perceived the impact and effects of progress testing in a free-response setting.

The intention was to hold two focus groups for each year group. Participants were recruited through student networks, with purposive sampling used to ensure representation across the different year levels and with different frequencies of exposure to progress testing. The focus groups were facilitated by a research assistant not involved in the medical programme. Ethics approval was granted by the University of Auckland Human Participants Ethics Committee.

Focus groups were recorded using a digital recorder and transcribed by an external contractor. Data from the transcripts were coded and sorted into categories, then arranged into themes using cross-sectional thematic analysis [15] by the primary researcher. The process of categorising the data and formation of themes was cross-checked by two other members of the research team. In addition an academic staff member independent of the research, with a good knowledge of progress testing, was invited to read the transcripts and undertake a thematic analysis independently of the researchers. This process elicited primary and secondary themes very closely aligned to those formed by the primary researcher. A ‘best-fit’ was achieved by consensus between the four people involved. From the themes, unifying constructs were identified and theory generated, using an inductive approach, and compared with the literature on progress testing. The key findings are presented in the following section, and compared with existing literature in the discussion.

In undertaking the process of analysis we recognise both the cautions and the benefits of reflexivity as it relates to our understanding and interpretation of the students’ experiences. The way in which we relate to and ascribe meaning to their experiences will be impacted by our shared context as teachers and learners. However, the method described above was designed to limit bias as far as possible regarding emergent themes.

## Results

A total of eight focus groups were held with Years 2–5 of the programme. There were two groups for each of Years 2 and 3, three for Year 4 and one for Year 5 (due to clinical placements limiting student availability). The groups consisted of between four and 10 students.

**Fig. 1:**
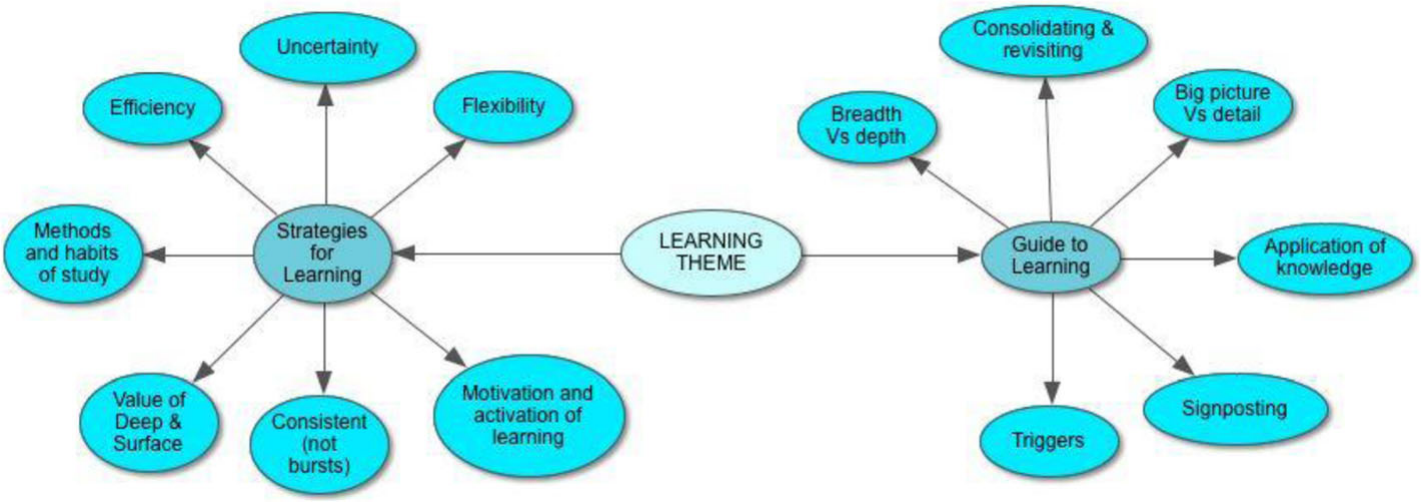
‘Impact on Learning’ Theme Diagram

**Fig. 2:**
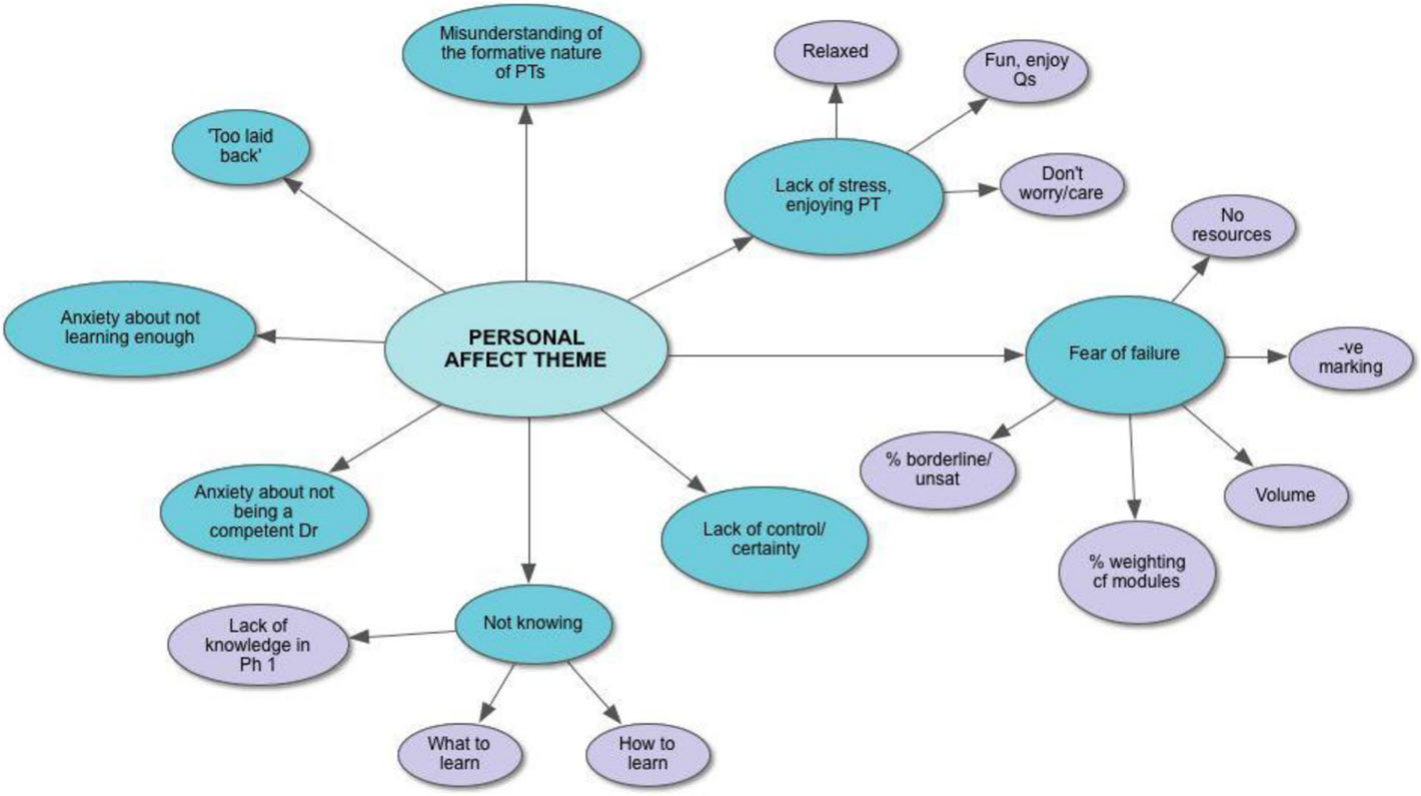
‘Emotional Wellbeing’ Theme Diagram

Three primary themes emerged from the data: ‘Impact on Learning’, ‘Emotional Wellbeing’ and ‘Operational Issues’. The first two of these were dominant and closely linked in the data, reflecting the study aims. Their sub-themes offer insights into student perceptions and behaviour (see Figs. 1 and 2). The key findings from these sub-themes are reported. Each of the themes will be outlined separately in this section, illustrated by quotations from the data, and then integrated within the broader discussion section. The third theme ‘Operational Aspects’ emerged as the students’ questions and concerns related to operational progress testing issues and are beyond the scope of this paper.

### Impact on learning

The students described the effects of progress testing on learning through a discussion of their own experience of preparing for, taking and processing the results of the progress tests.

#### Strategies for learning

Students identified a range of strategies that helped them to prepare for, and take, the progress test. A variety of habits were described, including consistency of study, the actual form their study takes, ways of being efficient and the need for flexibility. They were aware from briefings that intensive pre-test study for progress tests is not recommended, which appears to have driven them to find other approaches. Some students identified that it is more effective to work consistently towards the progress tests rather than trying to cram in bursts just before the tests, whereas others focused on other learning and assessments and ignored preparation for progress tests entirely.

I have noticed it’s encouraged me to study consistently throughout the year. I traditionally have been a big crammer, day before exams, and it’s helped motivate me to look up broadly everything the consultant might mention that I don’t know of, or, because it’s such a broad exam it’s forced me to study broadly and consistently [Yr 4 Gp 4].

My way of doing it is not to prepare. But I do research on the Internet or Wikipedia. Like as you learn more stuff you kind of look into that and research about clinical things [Yr 2 Gp 8].

They identified various methods of study, mostly focused on using clinically related search words and learning clinically applied material:

I study very differently to the normal lectures, the normal tests. I basically try to figure out things that might be very diagnostic and clinical straight off the bat [Yr 3 Gp 1].

I try and include the word clinical in my questions, or in notes I write so that when I search it before my test I’ll find them [Yr 3 Gp 7].

They note that their study patterns have changed from their previous reliance on factual memorisation:

So I find that, yeah I do a lot more of the reading study and not so much of the memorising and writing notes like formal study kind of thing. And I think it works [Yr 4 Gp 3].

I think you study less, but when you study you’re more aware of what you’re doing and whether it’s actually important. So you don’t cram or you don’t try and remember things that aren’t actually important [Yr 4 Gp 5].

Progress testing also encourages them to identify their knowledge limitations by the inclusion of a ‘don’t know’ response option. This provides good feedback on where they may need to focus future study.

I think it’s about studying smarter and knowing if you don’t know everything, knowing where the resources are, and I think progress tests reinforce that. Like yes, there’s a baseline body of knowledge you need to know to be a safe practitioner, but you also need to know what you don’t know and acknowledge that and decide whether or not you need additional resources [Yr 2 Gp8].

There was a difference noted between phases with respect to preparation for the tests. Those in Phase 2 noted that much of their learning is from their constant immersion in the clinical environment:

So to say I study less isn’t really true, because when I’m at hospital I’m engaged, I want to learn, even though I’m not necessarily studying you know? I wouldn’t say progress tests make you study less, it’s just different [Yr 4 Gp 3].

I think my study’s probably gotten a bit more efficient, ‘cause I’m definitely studying less… But then you can just focus more on the clinical stuff. So it is more in keeping with the way it’s taught on the wards as well as whatever teachings we have now [Yr 4 Gp 5].

For some students, identifying that there is no obvious or directed way to study is demoralising and confusing:

I usually study for exams and stuff, but I find study for this very overwhelming, because I really don’t know where to start. What topic to focus on. And even the feedback that we get, I don’t really use it because I feel like I need to study everything, but how can I study everything? [Yr 4 Gp 4].

Or the opposite:

I think it’s like really setting us up for the future as house officers and registrars, it’s not like you’ve got this test on this topic to study. It’s kind of like you’ve got this exam on everything and how you study each bit is up to you [Yr 4 Gp 3].

Although students did not commonly use the terms ‘deep’ or ‘surface’, they did discuss these different approaches in the way that they described their learning. Particularly after their first experience of progress tests, they identified some areas of knowledge where rote learning appeared to be valuable, but they also valued the deeper learning that comes from the integration and application of knowledge with their clinical experiences. In short, they indicated that they would adopt any strategy necessary to perform well.

Last year was really more like, “Oh, must get this grade.” Whereas now it’s more like, “I want to know more about it.” And I’ll actively go out and search for more content and stuff. I definitely wouldn’t have done that last year. I would’ve just been, what would have come up in the exam, and then studied for that [Yr 2 Gp 6].

‘Cause I’ll do a bit of study on theoretical stuff and then, because I have an idea of what the theory is, of course I can try and turn my brain into clinical mode and perhaps turn it into a progress test answer [Yr 3 Gp 7].

They also spoke about motivation, interest and curiosity in relation to progress tests. The cases presented seem to activate students’ learning. This engagement and activation was true for students in the clinical attachment years as well as Phase 1.

I think one good thing is that I’ve started to become more interested in learning things just because they’re interesting, not because they’re in a text book and you have to for your exam [Yr 4 Gp 4].

#### Guide to learning

Progress tests seem to provide triggers for learning. For example, students may see something in the clinical setting that reminds them of a question in a test, or they may recognise something in a question that they’ve seen before clinically. This provides reinforcement and enhances the learning process.

If you see a question in a progress test and you have no idea and then you learn about it later on or the next year, it might sort of spark something off in your memory about the progress test so it’ll make you remember that material better, and it helps things stick. So then when you see a patient with it for the first time clinically it might kind of feel like it’s not, because you’ve already experienced it through a test [Yr 3 Gp 1].

Sometimes the recognition is seen as partial or incomplete and has the potential to guide future learning. This occurs when students think they recognise something they know in a question, but then realise that in reality it relates to an application that they do not yet know (e.g. patient management or medication). This seemed particularly apparent in the Year 2 and 3 focus groups:

I find that in a lot of questions you’ll read it and you’ll know what the diagnosis is, and you’ve learnt stuff about the disease, but then you come to a question like what scan, or what drug, or where would you refer? It’s like we haven’t really been taught that. So we get some level of understanding of the question and then it just goes beyond what we’ve learnt [Yr 3 Gp 1].

As a consequence, in the early stages of the programme, students find that progress tests are useful for signposting future learning; that is, giving them an idea of what the important conditions are that they need to know about, since the tests are set at graduate level.

The fact that it was pitched at a graduate level and we’d been informed of that before time, left me quite relaxed and just curious more than anything to see what’s expected of us in time and seeing the sort of things that we might have to deal with [Yr 2 Gp 8].

Students talked about the sometimes uneasy tension between breadth and detail. They perceive that progress tests require them to use their knowledge and skills across a broad range of practice, encouraging a bigger picture view of medicine, whereas traditional written assessment sought a high degree of detail in smaller, more defined areas.

I think it does give a bit more perspective in that you kind of realise that the science stuff that we’re learning now, you kind of need to know and you need to understand it but you don’t really need to get too bogged down in the details [Yr 3 Gp 7].

This broad approach seems to be associated with the realisation that the aspects being tested are those that they really need to know to be a good practitioner:

And it’s like just being at the hospital that you slowly start thinking, “Oh okay, this patient comes in with chest pain, right, okay, these are the things I need to think about.” And in that regards the progress test is something that is good, because it kind of tests those need to know things… It kind of forces you to know those typical presentations and once you know those typical presentations, you can start adapting them to like untypical presentations [Yr 4 Gp 5].

There is strong evidence to indicate that progress tests point students in the direction of applying and integrating knowledge. In Years 2 and 3 (organ systems based learning) the tests help them to begin to apply their knowledge to clinical practice. When on clinical attachments in Years 4 to 6, they help them to apply their previous academic learning, including basic science, to practice.

The way in which we’ll be called upon to apply our knowledge outside of a very sort of sterile and artificial environment that you find in an exam, which is not something I do particularly well in. Having it set in a clinical context is quite nice [Yr 2 Gp 8].

However, for some, there is a perception that this focus on breadth is at the expense of the deeper knowledge that may be required. This was particularly pronounced with the Year 5 group, who were experiencing progress testing for the first time, following three years of traditional assessment methods. They saw a broad approach to learning as a negative aspect of progress testing, resulting in anxiety that they will not know everything they need for clinical practice.

I reckon, and I’m quite convinced that our generation of doctors are going to come through with quite significant gaps in our knowledge, because we haven’t been forced or encouraged to learn a lot of the things that we should know [Yr 5 Gp 2].

They note the importance of detail, and the lack of encouragement to learn it with progress testing, as compared to examinations held before their introduction.

I think no one’s going to sit down and learn the pharmacokinetics and dynamics for example, of morphine or propofol or something, unless they’ve actually got an exam that they have to learn it for. If you don’t learn it then, then you’re going to spend all your time in clinical practice when it’s being used just trying to learn it on the go, which isn’t a good way. It’s sometimes dangerous because you can get big gaps in your understanding... And I think now in retrospect those exams make you learn those details are important, because at least you’ve gone over it once [Yr 5 Gp 2].

The impact of progress tests on learning has been significant with respect to both strategies the students have developed for learning, and the way they can be used as a guide to learning. The data clearly portray progress testing as an assessment ‘for learning’, rather than simply ‘of learning’.

The second theme of ‘emotional wellbeing’ builds on these results in considering how progress tests have impacted on the students’ perceptions of their emotional responses, such as stress and anxiety, or, for some, enjoyment.

### Emotional wellbeing

The students described the effects of progress testing on their emotional wellbeing primarily through their experience of stress. They expressed the fear of failing, anxiety about negative marking, a feeling of lack of control, the worry of not learning enough, not knowing and therefore not becoming a competent doctor (see Fig. 2). Conversely some students spoke of enjoying progress tests and their lack of stress through not needing to study for them in the same way as traditional examinations.

The most negative effects expressed by the students related to feelings of being overwhelmed, anxiety, panic and anger; much of which focused on not yet knowing how to go about studying for the progress tests, not being sure of the implications of not doing well, and for some not understanding what the progress tests set out to assess and why.

The students from Phase 1 predominantly expressed stress about not knowing answers to many of the questions. It is to be expected that having been high achievers before acceptance into medical school they may find it difficult to initially score poorly in the progress tests. However some students appear to have gained a positive understanding of the purpose of the tests in the way that they are structured to test the outcomes of the programme.

I didn’t actually like it at the start because I found it made me feel quite useless because you have no idea what the questions are, and putting don’t know, don’t know, don’t know, is quite disheartening and discouraging at times. But as we go on I guess it’s quite apparent that you do know more and more as it goes on, which is quite encouraging progress. I guess that’s why it’s called a progress test [Yr 3 Gp 7].

Some also expressed the attitude of realising that initial low grades were to be expected and that there was therefore no point “stressing”:

I was kind of worried about failing it to be honest. ‘Cause I didn’t know much at all. But then again, I couldn’t do anything about it, so that’s why I was relaxed, because I couldn’t do anything. (laughter) Even though I was quite anxious that I might fail [Yr 2 Gp 6].

Across the years, the perception that there is a set percentage of students who will achieve an ‘unsatisfactory’ grade causes concern, and they identify how uncomfortable it would be for students to find themselves in either the borderline or unsatisfactory category:

I’m not saying I got in the bottom 5% but it would really suck for those couple of people who were in that bunch. Demoralising [Yr 4 Gp 4].

Similarly, while some students really worry about what they do not know, or feel angry that they’re being tested on things they haven’t been taught, others see this as a positive challenge:

At this stage it can be quite despairing when you look at it and you’re like, “I have absolutely no idea what any of this is, a lot of GP related questions, have not been taught this at all.” But I get quite excited about knowing that hey in about four years or three years I am going to know all this stuff… And I think that’s probably quite exciting that right now it looks like an insurmountable task, but in just a few short years we’ll be able to be like, “Hey we actually do know medicine.” [Yr 3 Gp 1].

Others express their emotions more as uncertainty than stress:

It’s like I don’t know how things are going to go. I don’t really have a good understanding of what’s going to come up in the test and that sort of thing. So I think for us, because it’s new, it’s a bit more uncertainty than it being more stressful [Yr 4 Gp 4].

Of those who felt significant stress about the progress tests, the increased weighting placed on the tests between Years 2 and 3 appears to contribute to this:

I think last year when it wasn’t worth much, it wasn’t much [stress], whereas this year it’s worth more than some of our module papers and that’s got a lot of people more stressed about it [Yr 3 Gp 1].

By the time they are in Years 4 and 5, 100% of the ‘Applied Science for Medicine’ grade is based on progress tests, which is perceived by some to add to their stress:

Adding to the problem is…if we do fail or show that we’re not progressing, to fail the whole year despite us doing well clinically I think that is just, yeah [Yr 4 Gp 4].

Whereas others expressed positive feelings about the tests in terms of raising their confidence level and alignment with their clinical experience:

Sometimes it’s quite cool, ‘cause it’s kind of more clinically based and sometimes when you are getting them right then you think, “Oh yeah, I can actually do this,” and that’s quite good. It gives you a bit of confidence for your clinical decision making, that’s a positive [Yr 4 Gp 4].

I think it’s better learning when you’re relaxed and genuinely interested and able to just like, I saw a cool patient so I’ll look that up at night. I don’t know whether it’s effective, but it’s more enjoyable [Yr 4 Gp 3].

Generally the Year 5 student group, in keeping with the ‘impact on learning’ theme, was more negative about the wellbeing aspects of progress testing, expressing concern that the progress tests have a detrimental effect on their knowledge:

I’m stressed about being a competent junior doctor. Because I don’t think I have the knowledge that doctors or house officers a couple of years ahead of us have, from having to learn all this, the curriculum [Yr 5 Gp 2].

This perception is in contrast to the majority of students who saw the progress tests as positively preparing them for practice (see previous findings).

Negative marking was also identified as a stressor by some, encouraging students to think in terms of probability rather than knowledge:

I find the negative marking thing quite stressful when I’m attempting the exam. I find myself having to strategically think about how I’m answering, which doesn’t really fit in with how I’m applying my knowledge [Yr 4 Gp 4].

…it has negative marking, it really encourages gambling. I’ve heard many of the students adopt the strategy that if you could eliminate one or two options off the multiple choice, you’re better off guessing. And I’ve actually done that and I’ve actually seen it work well. I dunno if that’s actually a good thing to do because, is that really learning? [Yr 3 Gp 7].

Overall, even for those who found progress tests to be stressful, they identified that they are less so than the traditional high-stakes end of year assessments.

I guess the really big factor is that because you do so many progress tests and it tracks your progress, it reduces the stress because you can bomb out on one and then you can have a couple of years and then bomb out on another one, which is nice to know that it’s not just one exam [Yr 4 Gp 5].

Speaking from purely a student point of view and not a future doctor point of view, I like them because it means you can’t cram for it, therefore you don’t cram for it. And there’s no point in stressing about it, because there’s nothing you can do, besides what you’re already doing. Whereas the fifth year exam used to be a very stressful event that everyone would really dread [Yr 5 Gp 2].

This leads us to be hopeful that the initial stress of progress testing for some students is outweighed by the ongoing stress and anxiety more generally associated with traditional examinations, where students commonly use ‘cramming’ as a study method.

## Discussion

In this study we explored some of the consequences of introducing progress testing from the perspective of students. The data was collected using focus groups during the first three years of introducing progress testing and included students from four years of the medical programme. Students described a variety of learning responses to progress testing; these were often constructive, sometimes strategic and occasionally negative. They see progress testing as guiding their learning, helping them to make connections between the science and practice of medicine. For junior students the content of progress tests acts as a signpost for future learning. For senior students they act as a reminder and reinforcer of core learning. Although some students talked about anxiety and their levels of stress, these were sometimes seen as triggers to learning and often related to their desire to perform well. In general there was a reduction in perceived stress as students became accustomed to the testing philosophy. Their comments about stress also supported the survey data results, where levels of stress were found to be significantly higher at the end of the year for students undergoing traditional end of year examinations of their applied medical knowledge [9].

Whilst the literature claims that progress tests promote deep learning, there does not appear to be empirical evidence to support this. The data from our survey [9] did not find significant changes to either deep or surface learning across the time points measured. However, the survey did find a high prevalence of existing deep learning, which may have influenced our ability to measure change. The focus group data suggests that both deep and surface approaches are being used strategically. Students are clearly employing a range of learning strategies based on their experience of sitting the progress tests, reviewing feedback, reflecting on their learning environment and looking forward to clinical practice. They seem to be identifying rote learning that is foundational (declarative knowledge) and deeper learning that helps them to solve clinical problems and to proactively apply knowledge in less familiar settings (procedural knowledge).

Wade et al. [16], in their study of student perceptions of progress tests across two schools of medicine in the UK, highlight the importance of context in the delivery of progress testing and the curriculum within which it sits. Our context fits somewhere between the two UK schools, but is aligned more with School B, with more frequent testing (three per year), provision of feedback and some early clinical learning. Our study expands on their findings, and provides further evidence for progress testing as being both assessment for learning and impacting the way students learn and study.

Students also identified the ways in which progress tests can guide their learning, through the contextualisation of test questions within clinical scenarios [2]. They speak of reinforcement, signposting, application and integration of their learning within the clinical environment.

Although anxiety and stress were words that students frequently used, the context was often related to lack of understanding of the purpose of progress tests and how to make strategic decisions in answering questions. In this respect, concerns may be short-lived, hopefully being alleviated with increasing experience in sitting the tests.

On examining the data for Year 5 as a whole rather than cross-sectionally, it was apparent that their perceptions of progress testing were more negative than the other year groups. It is acknowledged that change is unsettling and that students in transition may well ‘hang on’ to the familiar. The concerns of the Year 5 students are to be expected, given that they were the pioneers for multiple curricular changes in their medical programme and had also experienced a more traditional programme for two years before progress testing was introduced. Anxiety resulting from changes to assessment has been identified as a risk factor [17–19].

Student views were often polarised. For most of the concerns expressed by some students, other students experienced the opposite effect; see for example those students who indicated that they enjoyed progress tests precisely because they could not intensively cram for them and therefore experienced less stress. These differences most likely reflect either different personality types, or confidence relating to what year of the programme they were in. For example, in the early years students have reported stress in the context of achieving low grades. This has also been reported in the experience of other medical programmes internationally, for example, C. Ricketts (pers comm., Nov 15–19 2010) reported that the psychological effects of achieving very low scores in the first years needs to be mediated through appropriate induction, particularly for Type ‘A’ students and borderline students.

A limitation of the study is that although we collected data from four cohorts within our programme, providing some diversity, this was a single centre study and the findings may not be generalisable. However, we speculate that similar themes may be found when introducing progress testing in a similar setting. In addition, the survey data provides reassuring triangulation [9]. As the focus group question guide was based around learning approaches and stress, we may have missed some other unanticipated impacts of progress testing on student learning.

## Conclusions

This study provides qualitative evidence to support the assertion that progress testing influences the approach of medical students to their learning. We found that students employed a strategic mix of deep and surface approaches, and paid close attention to what they learned from the experience of sitting the tests. Although they valued the targeted feedback provided by learning points post-test, they learnt more broadly about integration, dealing with uncertainty and the context of current and future practice.

After initial uncertainty and anxiety, the majority of students in the study understood and supported the philosophy of progress testing, regardless of their individual approach to learning. Although these students experienced stress in relation to progress testing, this was sometimes constructed positively and seemed to reduce with exposure to testing. Where students compared it with traditional assessments, progress testing was viewed as no worse and sometimes better.

The fact that the Year 5 students tended to be more negative may be due to the mixed context of their curricular experience, moving from a traditional assessment environment to progress testing. This is perhaps similar to Wade et al.’s study where the programme that used infrequent testing alongside traditional tests was associated with more negative student views [16].

As a result of the findings we have continued to improve our preparation for the tests and the quality of post-feedback and targeted support. We have also ensured that the clinical tutors are well briefed on the format and purpose of the test, to reduce confusion generated in the clinical teaching environment.

Future research should focus on students who have become accustomed to progress testing since entry to the medical programme, examining learning approaches, stress levels and importantly the quality of graduate performance in the work place.

This study provides evidence that our goals for introducing progress testing have been largely met without undue additional stress. Students have clearly articulated the value of having an assessment for leaning, that signposts the importance of applied clinical knowledge, not only as undergraduates, but looking ahead to the early postgraduate years.
